# Investigation of the hypoglycemic mechanism of the ShenQi compound formula through metabonomics and 16S rRNA sequencing

**DOI:** 10.3389/fphar.2024.1349244

**Published:** 2024-04-19

**Authors:** Juan Gao, Xiujuan Zhou, Hong Gao, Guiping Xu, Chunguang Xie, Hongyan Xie

**Affiliations:** ^1^ Chengdu University of Traditional Chinese Medicine School of Clinical Medicine, Chengdu, China; ^2^ Hospital of Chengdu University of Traditional Chinese Medicine, Chengdu, China; ^3^ TCM Regulating Metabolic Diseases Key Laboratory of Sichuan Province, Hospital of Chengdu University of Traditional Chinese Medicine, Chengdu, China

**Keywords:** ShenQi compound, type 2 diabetes, gut microbiota, metabolomics, hypoglycemic mechanisms

## Abstract

**Introduction:** Herbal formulations are renowned for their complex biological activities, acting on multiple targets and pathways, as evidenced by *in vitro* studies. However, the hypoglycemic effect and underlying mechanisms of Shenqi Compound (SQ), a traditional Chinese herbal formula, remain elusive. This study aimed to elucidate the hypoglycemic effects of SQ and explore its mechanisms of action, focusing on intestinal flora and metabolomics.

**Methods:** A Type 2 diabetes mellitus (T2DM) rat model was established through a high-fat diet, followed by variable glucose and insulin injections to mimic the fluctuating glycemic conditions seen in diabetes.

**Results:** An eight-week regimen of SQ significantly mitigated hyperglycemia, inflammation, and insulin resistance in these rats. Notably, SQ beneficially modulated the gut microbiota by increasing populations of beneficial bacteria, such as Lachnospiraceae_NK4A136_group and Akkermansia, while reducing and inhibiting harmful strains such as Ruminococcus and Phascolarctobacterium. Metabolomics analyses revealed that SQ intervention corrected disturbances in Testosterone enanthate and Glycerophospholipid metabolism.

**Discussion:** Our findings highlight the hypoglycemic potential of SQ and its mechanisms via modulation of the gut microbiota and metabolic pathways, offering a theoretical foundation for the use of herbal medicine in diabetes management.

## 1 Introduction

Type 2 diabetes mellitus (T2DM) is a complex chronic metabolic disorder, predominantly characterized by sustained hyperglycemia and multi-organ dysfunction (Chatterjee et al., 2017). Approximately 537 million adults worldwide are affected by T2DM, which represents a leading cause of morbidity and mortality, accounting for nearly 6.7 million deaths in 2021 alone (Sun et al., 2022). A critical aspect of T2DM is glycemic variability, which has a broader meaning because it alludes to blood glucose swings from peaks to nadirs that occur over varying period (Suh and Kim, 2015; Zhou et al., 2020). Recent research indicates that glycemic variability, resulting from complex metabolic disturbances, significantly contributes to the onset of diabetes-related micro- and macroangiopathic complications (Domingueti et al., 2016; Kovatchev, 2017). Experimental evidence suggests that intermittent hyperglycemic peaks may accelerate the decline in insulin secretion and the functional deterioration of pancreatic islet cells more than constant high glucose levels (Del et al., 2007). Consequently, managing glycemic variability is crucial for minimizing diabetes-related complications.

Emerging evidence underscores the key role of the “host-metabolites-gut microbiota” axis, highlighting the central role of gut microbiota in mediating metabolic processes (Tripathi et al., 2018). The close interaction between liver-secreted metabolites and gut microbiota composition suggests a bidirectional relationship: metabolites produced by the gut microbiota can influence liver function while, in turn, the liver regulates gut microbiota growth through the secretion of metabolites (Milosevic et al., 2019; Albillos et al., 2020). A significant association exists between T2DM and imbalanced intestinal microbiota (Ringseis et al., 2020), where specific microbial metabolites, such as imidazole propionate and trimethylamine, have been implicated in insulin resistance and hepatic steatosis, respectively. Imidazole propionate can trigger insulin resistance by impairing mTORC1 signaling while trimethylamine can also induce hepatic steatosis (Sherriff et al., 2016; Koh et al., 2018). Conversely, fecal microbiota transplantation from lean to diabetic mice and the modulation of insulin signaling pathways by short-chain fatty acids (SCFAs), produced through microbial fermentation in the gut, have shown potential in improving body weight management and glycemic control (Remely et al., 2014; Rasmussen et al., 2020). These findings highlight the potentially pivotal role of gut microbiota in the pathogenesis of T2DM.

While numerous pharmacological interventions have been developed for T2DM management, traditional Chinese medicine (TCM) has demonstrated a unique capacity to address diabetes and its complications by exploiting historical knowledge and distinctive medical theories (Padhi et al., 2020). Multiple clinical trials have demonstrated that TCM can be used to reduce the risk of hypoglycemia and improve glycemic control in diabetes management (Ji et al., 2013). Shenqi compound formula (SQ) is one such TCM herbal formula, that is extensively employed for the management of diabetes. However, the specific effects of SQ on glycemic variability in T2DM and the underlying mechanisms, particularly through the perspective of intestinal flora and metabolomics, have not been fully elucidated. In this study, we set out to explore these aspects, contributing to a more comprehensive understanding of SQ in reducing glycemic variability and its potential as a natural remedy in the context of diabetes management.

## 2 Materials and methods

### 2.1 Materials

Rapid-acting insulin injections were sourced from Novo Nordisk Pharmaceuticals Co., Ltd. Hematoxylin-eosin (H&E) stain was purchased from Thermo Fisher Scientific (USA), and Oil Red O from Sigma-Aldrich (USA). Commercial kits for triglycerides (TG), total cholesterol (TC), high-density lipoprotein cholesterol (HDL-C), and low-density lipoprotein cholesterol (LDL-C) were acquired from MyBioSource, Inc. (China). Rat Tumor Necrosis Factor Alpha (TNF-α) and Rat Interleukin 6 (IL-6) ELISA Kits were obtained from Elabscience, Inc. (China).

### 2.2 Drug preparation

The eight herbal medicines constituting the various SQ formulations were procured from Sichuan Xinhehua Traditional Chinese Medicinal Herbs Co., Ltd. A detailed breakdown of the composition and constituents of SQ is provided in Table 1. The pharmaceutical preparation process was as follows: Panax ginseng C.A.Mey and other herbal components were separately soaked in purified water for 30 min. Subsequently, Panax ginseng C.A.Mey was boiled for 30 min. After this initial boiling phase, it was combined with the remaining herbal components. Upon reaching boiling, the mixture was vigorously boiled for an additional 40 min. The decoction was then filtered to obtain the medicinal liquid. The filtrate, obtained after two rounds of boiling, underwent distillation, concentration, and subsequent freeze-drying, resulting in a dried residue constituted the extraction material for SQ compound. This dried extract was stored at −20°C and thoroughly mixed with distilled water before use. A saxagliptin (SAX) suspension was prepared by dissolving saxagliptin tablets (Production License No.: J20160069; AstraZeneca Pharmaceuticals Ltd.) in distilled water.

**TABLE 1:** The composition and complete details of SQ.

No.	Chinese name	Botanical name	Family	Part used	Weight(g)	Batch number
1	Ren Shen	*Panax ginseng C.A.Mey.*	Araliaceae	Rhizome/root	15	2105132
2	Zhi Huang qi	*Aconitum Carmichael* Debx.	Leguminosae	Root	15	2105071
3	Sheng Di Huang	*Rehmannia glutinosa* (Gaertn).DC.	Scrophulariaceae	Root	10	2103068
4	Shan Yao	*Dioscorea oppositifolia* L.	Dioscoreaceae	Rhizome/root	10	2101021
5	Shan Zhu Yu	*Cornus officinalis* Sieb. Et Zucc.	Cornaceae	Fruit	10	2101063
6	Tian Hua fen	*Cuscuta chinensis* Lam.	Cucurbitaceae	Root	10	2102093
7	Dan Shen	*Salvia miltiorrhiza* Bunge.	Lamiaceae	Rhizome/root	10	2104111
8	Da Huang	*Radix Rhei Et* Rhizome	Polygonaceae	Rhizome/root	6	2104044

### 2.3 Animals and experimental design

Thirty-eight eleven-week-old male spontaneous type 2 diabetes Goto-Kakizaki (GK) rats (332–374 g) (bred from a Wistar strain background) and twelve age-matched male non-diabetic Wistar rats (365–379 g) were obtained from the Cavens Laboratory Animal Co., Ltd (Changzhou, Jiangsu; Permit No.: SCXK(Su)2016-0010). All experimental rats were housed under controlled conditions, at a constant temperature of 21°C ± 2°C, relative humidity of 60% ± 10%, and a 12/12-hour light-dark cycle, with *ad libitum* access to food and water.

The study employed four experimental groups: blank control (CON, *n* = 12), untreated diabetic model (MOD, *n* = 12), positive control (saxagliptin, 0.83 mg/kg body weight) (SAX, *n* = 13), and diabetes with medium-dose SQ (1.44 g/kg body weight) (SQ, *n* = 13). Following an adaptation period, the Wistar rats were fed a diet composed of 20% protein, 10% fat, 70% carbohydrates at 342 kcal/100g, over 10 weeks. To establish the T2DM model, GK rats were fed a high-fat diet, consisting of 88.2% standard feed, 10% refined lard, 1.5% cholesterol, and 0.3% pig bile salt. After 2 weeks of dietary manipulation, induction of T2DM in the GK rats was confirmed by random blood glucose levels that exceeded 11.1 mmol/L. To simulate glycemic variability in the T2DM rats, a regimen of staggered peak glucose and insulin intraperitoneal injections was employed. The rats were administered an intraperitoneal infusion of a 25% glucose solution at a volume of 0.375 mL/(kg∙d) at 10:00 AM. This was followed by a subcutaneous injection of NovoRapid insulin solution, at a dosage of 0.7 units per 100 g of body weight (U/100g), at 14:30 PM. The CON group received equivalent volumes of intraperitoneal injections of 0.9% saline and subcutaneous sham injections of the same intensity. The rats in the SAX and SQ groups were administered with the assigned compound via gastric gavage, once daily, throughout the eight-week intervention phase. Rats in the CON and MOD groups received equivalent volumes of purified water. At the end of the experiment, all rats underwent a 12-hour fast with *ad libitum* access to water before being sacrificed. Blood, liver, and fresh feces samples were collected and immediately stored at −80°C for subsequent analysis.

### 2.4 Evaluation of glycemic variability model

Weekly recordings of blood glucose levels were conducted at five time points (8:30 AM, 10:30 AM, 14:30 PM, 16:30 PM, and 18:30 PM) to determine the glycemic fluctuations. Several parameters, including the largest amplitude of glycemic excursions (LABG), daily mean blood glucose (MBG), and the standard deviation of daily average blood glucose (SDBG), were employed to evaluate day-to-day glycemic variability among the different groups. The success of the diabetes glycemic variability model induction was confirmed in line with expert consensus (Danne et al., 2017), which recommends SDBG ≥2.0 mmol/L and LABG ≥4.4 mmol/L.

### 2.5 Measurement of fasting blood glucose (FBG), oral glucose tolerance test (OGTT), and fasting insulin in serum

Animal body mass and FBG were determined. The OGTT was conducted at the end of the experiment, and involved a 14 h fast, followed by an oral 2.0 g/kg dose of glucose with pre- and post- (0, 30, 60, and 120 min post glucose) blood glucose quantification via a retro-orbital bleed and glucometer. The fasting insulin in serum (FINS) levels were determined using ELISA kits. The homeostasis model assessment-insulin resistance (HOMA-IR) was calculated via the following formula: The HOMA-IR = [fasting blood glucose (mmol/L)] × [fasting insulin (μIU/mL)]/22.5.

### 2.6 Determination of biochemical and inflammatory factor level analysis

Biochemical serum lipid profiles, including TC, TG, HDL-C, and LDL-C, were quantified using a fully automated biochemical analyzer (Hitachi). In addition, specific ELISA assay kits were used to quantify TNF-α and IL-6 levels in plasma.

### 2.7 Histopathological examination

Anesthesia was performed by an intraperitoneal injection of 20% urethane solution at 0.5 mL/100g body weight. Immediately, fresh hepatic tissues were collected and weighed to calculate the liver organ index using the formula: Liver Mass/Body Weight × 100%. A portion of these liver tissues was fixed in 4% paraformaldehyde for 48 h and subjected to H&E staining for histological analysis. Histological images of the hepatic tissues were captured using an optical microscope (Leica Microsystems, Japan). Five frozen liver samples from each group were flash-frozen at −80°C for 48 h to prepare them for oil red O staining. Three tissue sections were selected from each sample to observe the aggregation of lipid droplets (red signal) and the changes in the positive signal in the liver under an optical microscope. Image Pro Plus 6.0 software was used to calculate the area of the positive oil red O signal.

### 2.8 16S rRNA sequencing

16S rRNA sequencing employed a fecal genomic DNA kit (Tiangen et al., China). DNA extraction was performed by adopting the CTAB/SDS method from the colon contents samples. The amplification of the V3 and V4 hypervariable regions of the 16S rRNA gene was performed using specific primers, 515F and 806R. Subsequently, the sequencing library was constructed by employing the TruSeq DNA PCR-Free Sample Preparation Kit (Illumina, USA), and high-throughput sequencing was performed on the NovaSeq6000 sequencing platform. The bioinformatics analysis of the resulting microbiome data was carried out using QIIME 2 software.

### 2.9 Untargeted liver metabolomic analysis

Hepatic tissue samples (100 g) were homogenized and combined with a pre-chilled methanol/acetonitrile mixture (volume ratio 1:1) in Eppendorf (EP) tubes. Following thorough agitation and ultrasonication in an ice-water bath for 10 min, the samples underwent high-speed cold centrifugation at 12,000 g for 15 min at 4°C. Subsequently, 500 μL of the supernatant was extracted and placed in EP tubes, then dried in a vacuum concentrator. The dried extracts were reconstituted with 160 μL of a water/acetonitrile mixture (volume ratio = 1:1) and centrifuged at 12,000 g for 15 min at 4°C. The resulting supernatant was then subjected to liquid chromatography-mass spectrometry (LC-MS).

Metabolomic analysis was conducted using an ultra-high-performance liquid chromatography (UPLC) system (Waters Acquity I-Class, China) coupled with a quadrupole time-of-flight (QTOF) mass spectrometer (Waters Xevo G2-XS, China). Analytical separation was achieved using a Waters UPLC HSS T3 column (2.1 × 100 mm, 1.8 μm). The UPLC system was operated at a constant flow rate of 0.4 mL/min, with an injection volume of 1 μL for gradient elution. Eluent A (0.1% formic acid) and eluent B (0.1% formic acid in acetonitrile) were employed in positive ion mode. The cone voltage was set as 30V, and the ion source temperature was maintained at 100°C. Desolvation gas flow was set at 800 L/h. An auxiliary gas heating temperature of 500°C and a counter-flow gas rate of 50 L/h were applied.

Raw data acquired from the UPLC-MS were analyzed using the Masslynx 4.2 code. Before further analysis, the raw data underwent preprocessing by employing the Progenesis QI code. Metabolite identification was conducted by referencing the METLIN database and a custom library, followed by data normalization. Principal component analysis (PCA) and orthogonal projections to latent structures-discriminant analysis (OPLS-DA) were performed within the SIMCA-P+ software to further refine and select differential metabolites. Volcano plots were constructed by utilizing the ggplot two package in the R programming language. The correlation analysis between metabolites and differential microbial communities was conducted using the Pearson correlation coefficient.

### 2.10 Statistical analysis

Quantitative data were analyzed and presented as mean ± standard deviation. Statistical data analysis was conducted using SPSS 25.0, using one-way analysis of variance (ANOVA) and Student’s t-tests. Significance thresholds were set at *p* < 0.05 and *p* < 0.01, denoting different levels of confidence.

## 3 Results

### 3.1 Evaluation of the glycemic variability model

**FIGURE 1:**
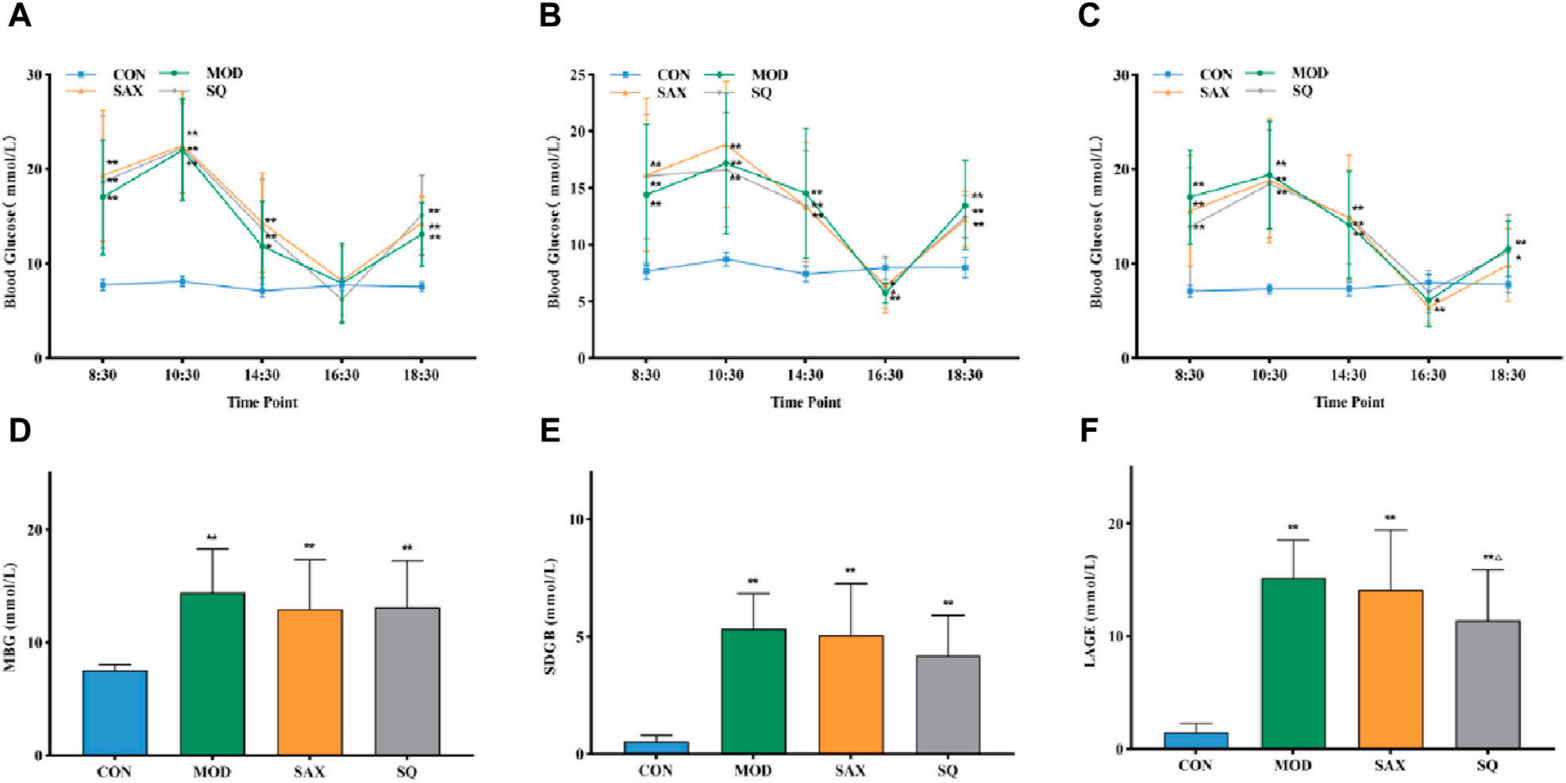
Daily blood glucose trends in rats. **(A)** Third week, **(B)** Sixth week, **(C)** Tenth week, **(D)** MBG, daily mean blood glucose in the tenth week, **(E)** SDBG, standard deviation of daily average blood glucose in the tenth week, **(F)** LABG, the largest amplitude of glycemic excursions in the tenth week. Data are presented as mean ± SD. *(*p* < 0.05), and **(*p* < 0.01) vs. CON; ^Δ^(*p* < 0.05), and ^ΔΔ^(*p* < 0.01) vs. MOD. CON (*n* = 12), blank control group; MOD (*n* = 12), untreated diabetic model group; SAX (*n* = 13), positive control group; SQ (*n* = 11), diabetes with medium-dose SQ group.

To assess glycemic variability during the modeling phase, the average blood glucose fluctuation trend was monitored at five specific time points within 24 h at the third, sixth, and tenth weeks. As illustrated in Figure 1 A-C, the GK rats exhibited significant peaks and troughs in blood glucose levels over 24 h, in sharp contrast to the stable blood glucose levels of the CON group. Additionally, elevated blood glucose levels in the MBG, SDGB and LAGE groups among diabetic rats (Figures 1D–F) confirmed the successful establishment of the diabetic glycemic variability model.

### 3.2 Effects of SQ on glucose metabolism and insulin sensitivity

**FIGURE 2:**
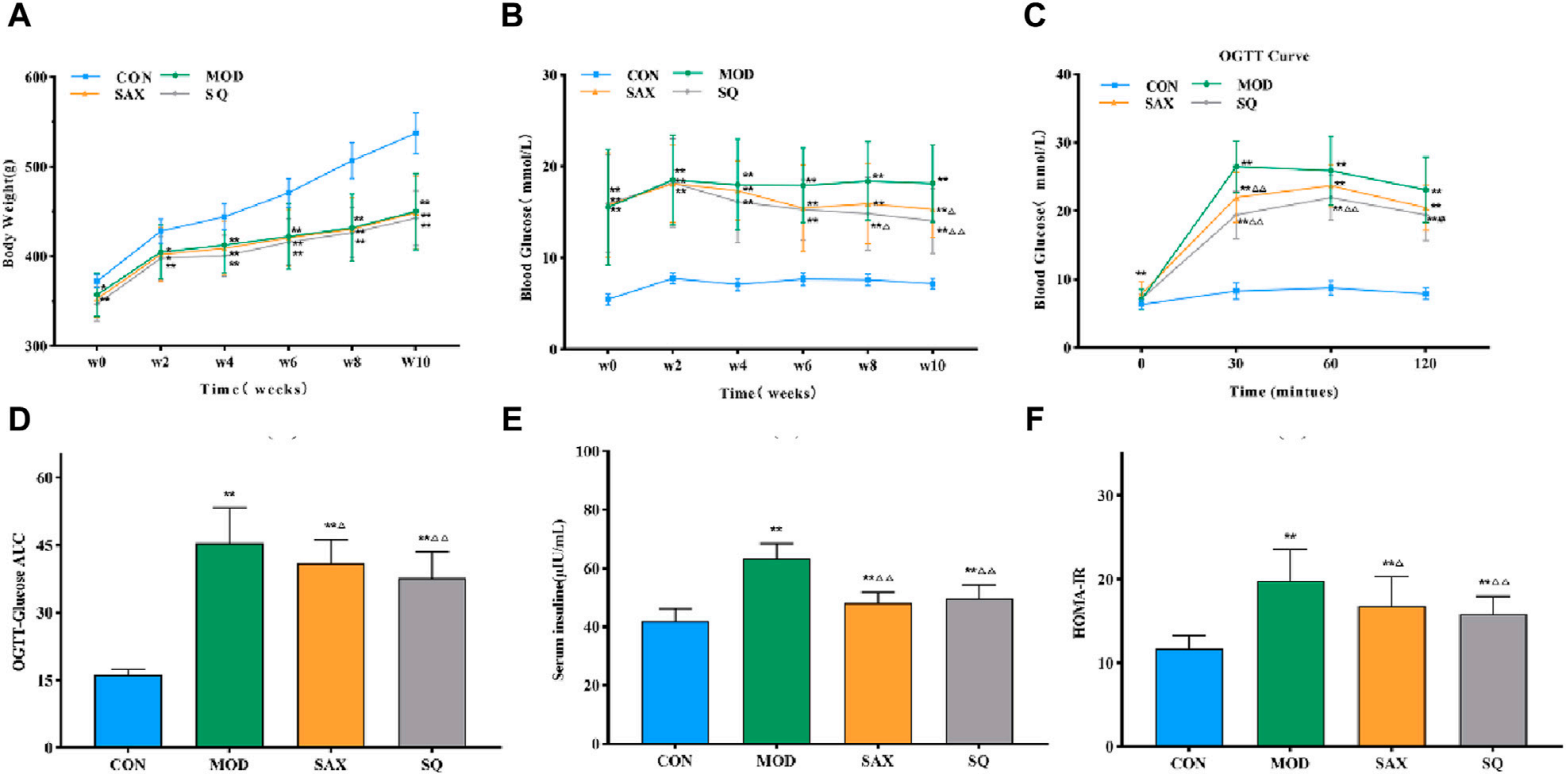
SQ effects on glucose metabolism and insulin sensitivity in T2DM. **(A)** Body Weight, **(B)** FBG, **(C)** OGTT, **(D)** OGTT-AUC, **(E)** FINS, **(F)** HOME-IR. Data are presented as mean ± SD. *(*p* < 0.05), and **(*p* < 0.01) vs. CON; ^Δ^(*p* < 0.05), and ^ΔΔ^(*p* < 0.01) vs. MOD. CON (*n* = 12), blank control group; MOD (*n* = 12), untreated diabetic model group; SAX (*n* = 13), positive control group; SQ (*n* = 11), diabetes with medium-dose SQ group.

Following the establishment of the T2DM model, diabetic rats displayed symptoms of polydipsia, polyphagia, polyuria, lean body shape, weakened reaction, dull hair, and local skin rash. These metabolic phenotypes were further characterized. Figures 2A, B show that, despite similar food intake, diabetic GK rats experienced a gradual increase in body weight and higher blood glucose levels compared to the CON group. Intervention with SAX and SQ did not significantly affect body weight but did lower blood glucose levels in T2DM rats in later stages of the experiment, with reductions in FBG levels by 16.02% and 22.65%, respectively.

Additionally, compared to the CON group, diabetic rats demonstrated reduced glucose tolerance via OGTT (Figures 2C, D), increased insulin levels (Figure 2E), and higher HOMA-IR (Figure 2F), indicating marked insulin resistance. SAX and SQ treatments improved glucose tolerance in OGTT. Meanwhile, the serum FINS levels in the SAX and SQ groups decreased by 23.97% and 21.61%, respectively, along with a significant reduction in HOMA-IR values, indicating that these treatments regulated blood glucose homeostasis in diabetic rats.

### 3.3 Effects of SQ on serum biochemistry and inflammatory factors

**FIGURE 3:**
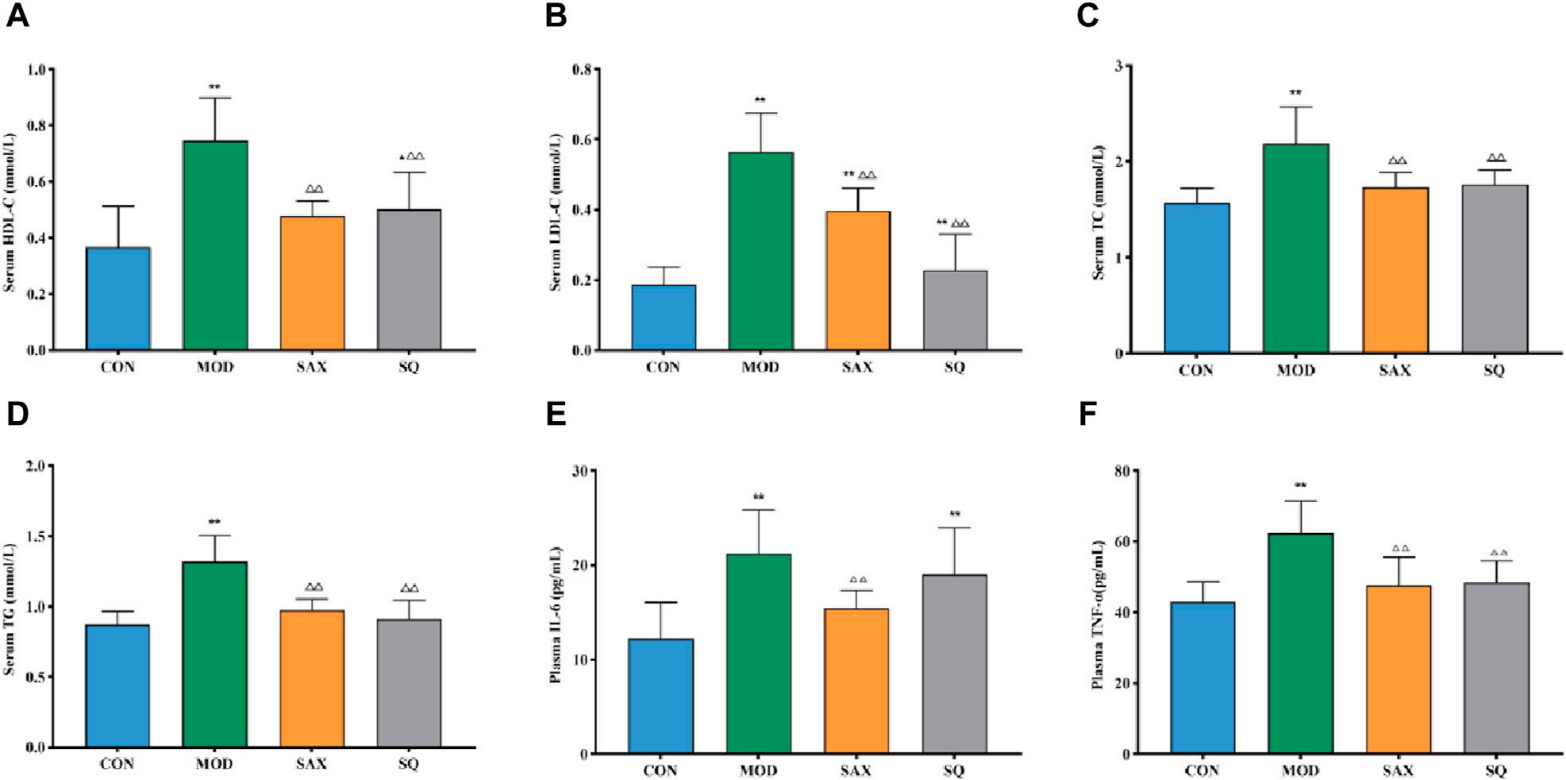
SQ effects on serum biochemistry and plasma inflammatory factors. **(A)** HDL-C, **(B)** LDL-C, **(C)** TC, **(D)** TG, **(E)** IL-6, and **(F)** TNF-α. Data are presented as mean ± SD. *(*p* < 0.05), and **(*p* < 0.01) vs. CON; (*p* < 0.05), and (*p* < 0.01) vs. MOD. CON (*n* = 8), blank control group; MOD (*n* = 8), untreated diabetic model group; SAX (*n* = 8), positive control group; SQ (*n* = 8), diabetes with medium-dose of SQ group.

Given the close link between dyslipidemia, chronic low-grade inflammation, and insulin resistance (Jaiswal et al., 2014), a comparative analysis of serum biochemical parameters (HDL-C, LDL-C, TC, TG) and plasma inflammatory factors (IL-6, TNF-α) was conducted. In the MOD group, diabetic rats exhibited significantly elevated levels of TC, TG, HDL-C, and LDL-C in serum (Figures 3A–D), along with increased levels of IL-6 and TNF-α in plasma (Figures 3E, F), signifying the presence of dyslipidemia and a severe inflammatory response induced by the high-fat diet. Compared to the MOD group, SAX and SQ interventions significantly reduced the serum levels of HDL-C, LDL-C, TC, TG, and plasma levels of TNF-α in the T2DM rats. Additionally, SAX treatment notably decreased plasma IL-6 levels. These outcomes suggest that SQ has a beneficial effect in correcting blood lipid abnormalities and mitigating inflammatory responses in diabetic rats.

### 3.4 Histopathological analysis

**FIGURE 4:**
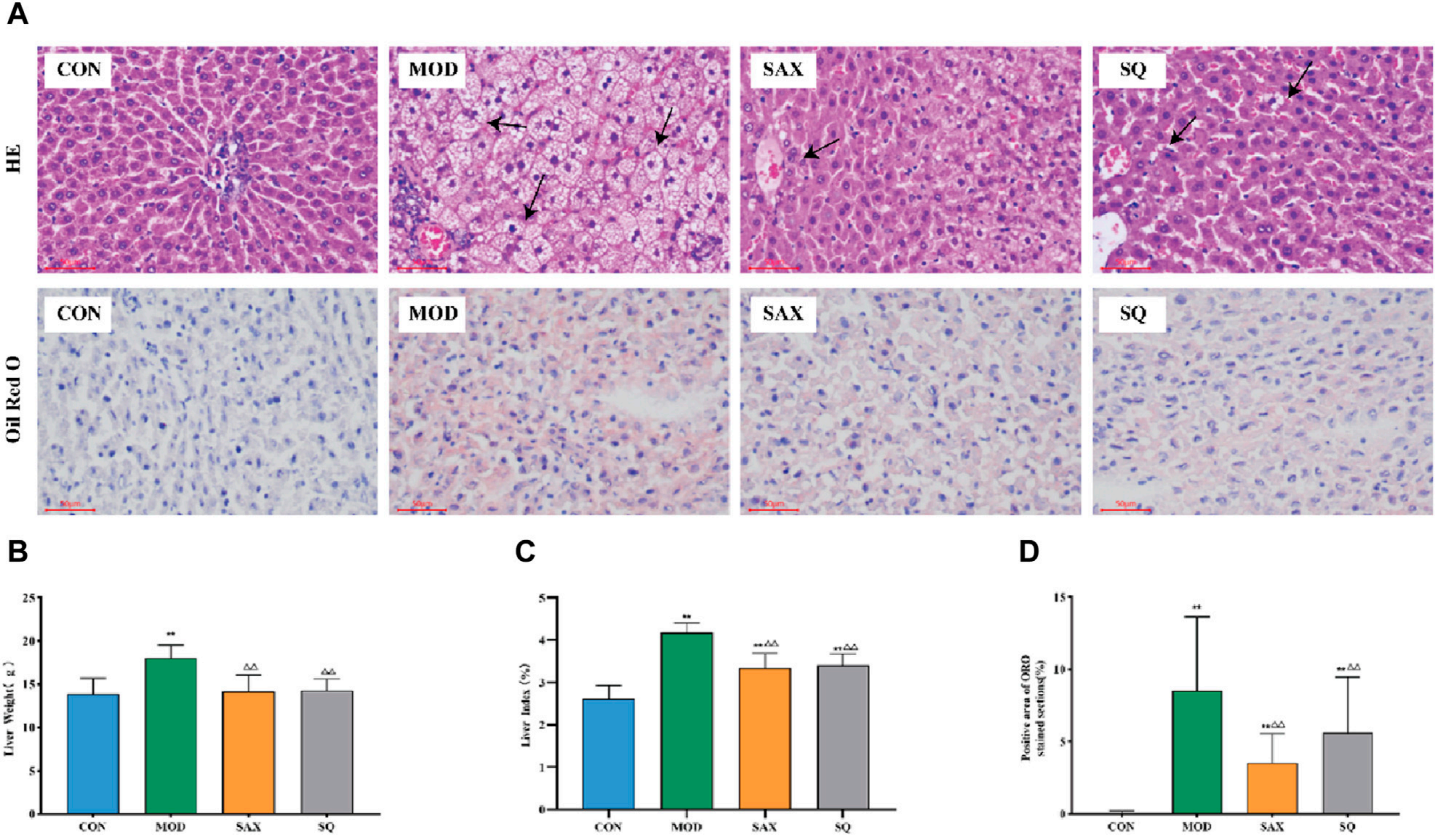
Histopathological changes in the liver of diabetic rats. Bar = 50 μm. **(A)** H&E and ORO, **(B)** Liver weight, **(C)** Liver index, **(D)** Positive area of ORO histopathological changes. Data are presented as mean ± SD. * (*p* < 0.05), and **(*p* < 0.01) vs. CON; (*p* < 0.05), and (*p* < 0.01) vs. MOD. CON (*n* = 8), blank control group; MOD (*n* = 8), untreated diabetic model group; SAX (*n* = 8), positive control group; SQ (*n* = 8), diabetes with medium-dose of SQ group.

The liver, critical for lipid metabolism, can be adversely affected by excessive fat intake. As illustrated in Figure 4A, in the MOD group, histological analysis revealed structural damage and lipid degeneration, as evidenced by small vacuoles within the cytoplasm. This damage is likely attributable to the direct impact of the high-fat diet on hepatic cells. Additionally, oil red O staining revealed numerous lipid droplets, accompanied by increased liver weight and liver coefficient in the MOD group (Figures 4B–D), providing further evidence of disordered hepatic lipid metabolism. Following intragastric administration of SAX and SQ, liver wet weight and coefficient were significantly reduced, and oil red O staining indicated fewer and smaller lipid droplets in the liver, demonstrating the efficacy of SQ in reducing lipid accumulation.

### 3.5 Effect of SQ on the fecal microbiota

**FIGURE 5:**
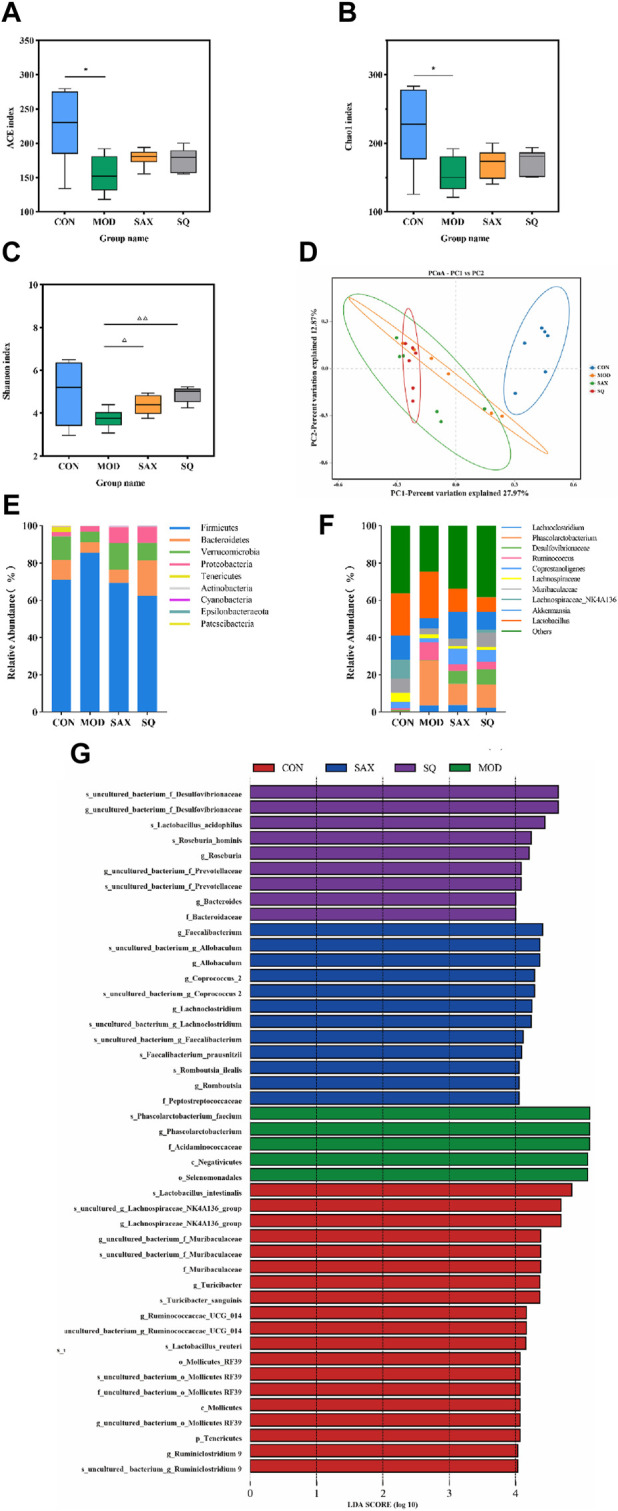
SQ effects on the fecal microbiota in T2DM rats. Alpha Diversity, including **(A)** ACE index, **(B)** Chao1 index, **(C)** Shanon index, **(D)** PCA diversity, **(E)** Colon microbiota composition at the phylum, **(F)** Colon microbiota composition at the genus levels, **(G)** LEfSe analysis. Data are presented as mean ± SD. *(*p* < 0.05), and **(*p* < 0.01) vs. CON; (*p* < 0.05), and (*p* < 0.01) vs. MOD. CON (*n* = 6), blank control group; MOD (*n* = 6), untreated diabetic model group; SAX (*n* = 6), positive control group; SQ (*n* = 6), diabetes with medium-dose of SQ group.

Alpha diversity analysis explored changes in bacterial diversity and abundance, as illustrated in Figures 5A–C. The ACE, Chao1, and Shannon indices collectively demonstrated a notable decrease in microbial diversity and abundance in the MOD group compared to the CON group. Following interventions with SAX and SQ, an increase in bacterial diversity and abundance was observed. Beta diversity analysis, aimed at assessing the overall structural changes in the gut microbiota, utilized principal Coordinate Analysis (PCoA). The analysis showed that the first two axes (PCo1 and PCo2) accounted for 27.97% and 12.87% of the total variance, respectively, indicating significant separation and distinct clustering among the samples (Figure 5D).

At the phylum level, intervention with SAX and SQ significantly altered the relative abundance of Firmicutes and Bacteroidetes, resulting in a decreased Firmicutes/Bacteroidetes (F/B) ratio, which is considered indicative of a healthier composition of the gut microbiome (Figure 5E). At the genus level, compared to the CON group, the MOD group exhibited higher relative abundances of Ruminococcus_1 and Phascolarctobacterium, along with lower levels of Akkermansia and Lachnospiraceae_NK4A136_group. Following treatment with SAX and SQ, these imbalances were corrected, shifting towards the bacterial profiles observed in the CON group, suggesting a normalization of gut microbiota (Figure 5F).

Linear Discriminant Analysis Effect Size (LEfSe) analysis, employing the Linear Discriminant Analysis (LDA) effect size, identified significant biomarkers with an LDA score >4, indicating substantial differences in observed variations. This analysis identified species such as Roseburia_hominis and *Lactobacillus*_acidophilus as biomarkers that are significantly associated with the SQ treatment groups. These outcomes underscore the potential of SQ to beneficially modulate the gut microbiota composition in diabetic rats by reducing the relative abundance of pathogenic bacteria such as Ruminococcus and enhancing beneficial bacteria, notably Roseburia and *Lactobacillus* (Figure 5G).

### 3.6 Effects of SQ on the liver metabolome

**FIGURE 6:**
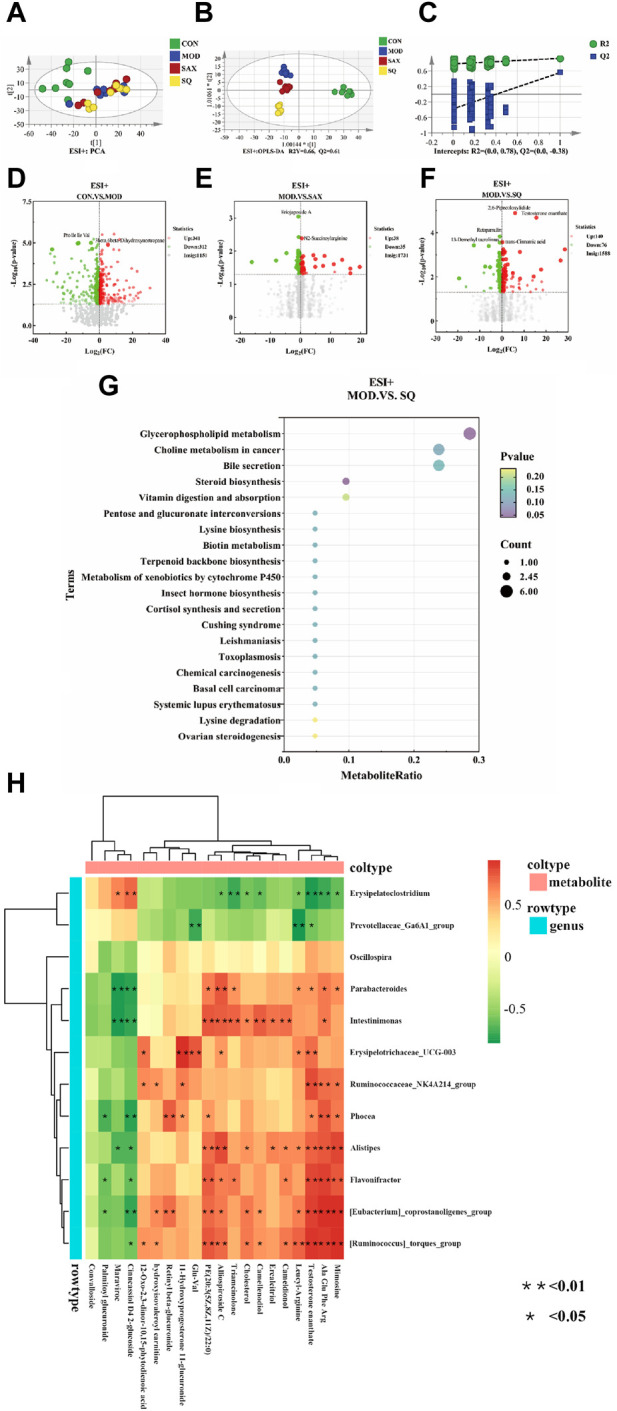
SQ effects on hepatic metabolic profiling. **(A)** PCA analysis, **(B)** OPLS-DA analysis, **(C)** OPLS-DA permutation test, Volcano plot (**(D)**, CON vs. MOD; **(E)**, MOD vs. SAX; and **(F)**, MOD vs. SQ), **(G)** Metabolic pathway enrichment, **(H)** Correlation analysis between microorganisms and metabolites. CON (*n* = 8), blank control group; MOD (*n* = 8), untreated diabetic model group; SAX (*n* = 8), positive control group; SQ (*n* = 8), diabetes with medium-dose of SQ group.

An untargeted metabolomic approach was utilized to explore the impact of T2DM with glycemic variability on liver metabolism. The distribution of hepatic metabolic profiles between the diabetic and control groups showed conspicuous separation, indicating significant alterations in liver metabolites associated with metabolic dysregulation within the T2DM rat model (Figure 6A). Our OPLS-DA analysis further validated this, with the score scatter plots exhibiting clear separation for the MOD and SQ groups, emphasizing the metabolic impact of SQ treatment (Figure 6B). Permutation tests for the PLS-DA model, conducted 200 times, demonstrated the reliability of the model by showing that all blue Q^2^ points were positioned below the green R^2^ point from left to right, confirming the model’s validity and the absence of overfitting (Figure 6C). Metabolites related to the progression of T2DM were identified using a combination of Variable Importance in Projection (VIP) scores and *p*-values or fold change (FC) values from a univariate analysis. Volcano plots highlighted differentially regulated metabolites as potential biomarkers between the groups. Specifically, biomarkers differentiating the CON and MOD groups included 3beta-6beta-Dihydroxynortropane and Pro Ile Ile Val, while Eriojaposide A and N2-Succinoylarginine were identified between the SAX and MOD groups (Figures 6D,E). 91 metabolites distinguished the SQ group from the MOD group, with significant alterations in metabolites such as trans-Cinnamic acid and Testosterone enanthate, highlighting their potential as biomarkers of the therapeutic effect of SQ (Figure 6F, Supplementary Table S1).

A bubble map, based on the KEGG pathway annotation of regulated metabolites, was used to explore the potential metabolic pathways impacted by SQ treatment (Figure 6G). The results indicated enrichment in 38 metabolic pathways following SQ intervention. This enrichment was particularly notable in Glycerophospholipid metabolism, followed by other pathways such as Choline metabolism in cancer, Bile secretion, Steroid biosynthesis, and Vitamin digestion and absorption. These metabolic pathways provide further evidence for the potential of SQ to improve glycemic variability in diabetes.

### 3.7 Effect of SQ on correlation analysis between microorganisms and metabolites

A correlation heatmap was utilized to visually depict the strength and direction of associations, aiming to uncover potential functional connections between intestinal bacteria and liver metabolism. As shown in Figure 6H, the analysis indicated that the relative abundances of Flavonifractor and Alistipes were positively correlated with Testosterone enanthate. In summary, SQ exhibited the capacity to influence the composition of the gut microbiota, thereby contributing to improvements in hepatic metabolite concentrations.

## 4 Discussion

Herbal medicines, with their multi-targeted, multi-pathway biological activities, have garnered increasing attention for their anti-inflammatory, anti-obesity, and hypoglycemic effects (Xie and Du, 2011; Xu et al., 2018). Specifically, the hypoglycemic effects of Chinese herbal drugs and formulations, including SQ, have been attributed to improvements in the gut microbiota and metabolic profiles in diabetic models (Zhong et al., 2022). However, the exploration of these effects has predominantly focused on individual genes or protein-related signaling pathways, leaving a comprehensive assessment of the mechanisms underlying TCM formulations, including SQ, largely unexplored. This study aims to fill that gap by elucidating the hypoglycemic activity and mechanisms of SQ, thereby contributing to the advancement of TCM as a supplementary therapeutic approach for diabetes prevention and management.

T2DM is characterized by hyperglycemia, insulin resistance, and dyslipidemia. Correspondingly, the MOD group showed elevated levels of FBG, OGTT, FINS, and HOMA-IR. Remarkably, SQ treatment reversed these markers, indicating its efficacy in alleviating hyperglycemia and insulin resistance. Additionally, SQ treatment modified serum levels of TC, TG, HDL-C, and LDL-C, and significantly reduced plasma TNF-α levels in rats, highlighting its potential in lipid metabolism regulation and glucose homeostasis maintenance. The elevation of serum lipid metabolism may contribute to aberrant hepatic lipid metabolism, an idea that was supported by the findings of our hepatic histopathological analysis which revealed small round vacuoles and lipid droplets in the liver cells of the MOD group. Remarkably, SQ treatment significantly ameliorated the hepatic tissue damage in diabetic rats and reduced the number of lipid droplets within the liver, highlighting its potential role in regulating blood glucose homeostasis.

The intestinal microbiota plays a crucial role in regulating host metabolism and is integral to maintaining blood glucose homeostasis (Ramakrishna, 2013). Previous studies have indicated that diabetic conditions are often accompanied by diminished diversity and evenness within the gut microbial community (Le Chatelier et al., 2013), findings that align with our observations. However, SQ treatment was found to enhance α-diversity, indicating an improvement in microbial community diversity that counteracts the adverse effects observed in diabetic rats. Moreover, analysis of β-diversity revealed that SQ intervention could effectively improve the overall structure of the intestinal microbiota in diabetic rats. Notably, at the phylum level, the Firmicutes-to-Bacteroidetes (F/B) ratio, often linked with obesity and insulin resistance (Hung et al., 2021), was reduced following SQ treatment, signaling a potential shift towards a healthier gut microbiota composition. At the genus level, SQ treatment markedly increased the abundance of beneficial bacteria such as Akkermansia and Lachnospiraeae_NK4A136, known for their positive impacts on metabolic health (Zhao et al., 2017; Zhou et al., 2022). Moreover, Ruminococcus has been implicated in triggering inflammatory responses within the immune system via oxidative stress mechanisms (Gurung et al., 2020; Mokkala et al., 2021), while Phascolarctobacterium has been observed to have a significant positive correlation with FBG levels, acting as a predictive marker for diabetes (Yang et al., 2021). The SQ intervention was effective in reducing the abundance of Ruminococcus and Phascolarctobacterium, which are considered conditional pathogens associated with inflammation, obesity, and diabetes. Additionally, LEfSe analysis confirmed Roseburia_hominis and *Lactobacillus*_acidophilus as distinctive biomarkers within the gut microbiota of the SQ-treated group, based on the LDA scores. Prior studies have highlighted the roles of *Lactobacillus*_acidophilus and Roseburia_hominis in mitigating intestinal epithelial dysfunction, dampening metabolic inflammation, and correcting intestinal dysbiosis, thereby aiding in the management of obesity and disorders of glucose metabolism (Yao et al., 2020; Tang et al., 2021; Kang et al., 2022). Previous studies also have indicated that SQ could ameliorate T2DM by reduce inflammation and improve intestinal barrier damage (Zhang et al., 2022). Thus, SQ treatment was shown to effectively modulate the diversity and structure of the intestinal microbiota, augmenting the presence of beneficial bacteria and diminishing the pathogenic bacteria. This modulation represents a key mechanism through which SQ exerts its hypoglycemic, insulin sensitivity-enhancing, and anti-inflammatory effects, exhibiting its multifaceted approach to combating diabetes and associated metabolic disturbances.

The liver is integral to the body’s physiological metabolism, significantly influencing the alterations of hepatic metabolic biomarkers. After SQ intervention, metabolomic analysis showed notable increases in secondary metabolites such as Trans-cinnamic acid and Testosterone enanthate. Previous research has demonstrated the role of Trans-cinnamic acid in promoting the browning of white adipocytes, offering a therapeutic avenue for obesity management and reduction of fat accumulation and hepatic lipid deposition (Kang et al., 2019; Wang et al., 2020). Concurrently, Testosterone enanthate has been shown to be effective in diminishing fat accumulation and improving insulin resistance in males with T2DM (Hackett, 2019). The examination of metabolic pathways impacted by these differential metabolites aids in delineating the mechanisms of SQ hypoglycemic action in diabetic models. Notably, pathways such as glycerophospholipid metabolism have been identified to modulate insulin resistance and lipid metabolism, thereby mitigating cardiac damage in models of diabetic cardiomyopathy (Dong et al., 2021; Jiang et al., 2022). Choline metabolism plays a crucial role in the liver, regulating critical genes involved in gluconeogenesis in hepatic cells to improve glucose metabolism (Chandler and White, 2019). Overall, it appeared that SQ could reduce fat accumulation and improve insulin resistance by regulating secondary metabolites and metabolic pathways to alleviate diabetic symptoms.

Alterations in the intestinal microbiota triggered by aberrant glucose and lipid metabolism lead to host insulin resistance, low-grade inflammation, and fat accumulation (Boulangé et al., 2016). Integrating metabolomic with LEfSe analysis, the observed heatmap correlation indicated that genera Alistipes and Flavonifractor, which increased following SQ treatment, positively correlated with Testosterone enanthate. These findings underscore the therapeutic potential of Alistipes in mitigating colitis severity in mice, emphasizing its regulatory role in immune responses through the modulation of anti-inflammatory cytokines (Dziarski et al., 2016). Flavonifractor, potentially linked with the dietary inflammation index, has been shown to inhibit IL-17 signaling in leukocytes, thereby attenuating intestinal inflammation (Mikami et al., 2021; Lozano et al., 2022). In summary, SQ has the potential to alter the composition of intestinal microbiota, particularly in relation to intestinal inflammation, thereby contributing to the improvement of the hyperglycemic profile in T2DM.

The complexity and individual variability of human gut microbiota complicate the elucidation of host-microbe interactions. Rodent models, especially those replicating diet-induced metabolic disorders akin to diabetes, offer valuable insights into the human condition (Preguiça et al., 2020). These models are critical in assessing the impact of microbiota composition changes on diabetes (Roesch et al., 2009), promoting a deeper understanding of the role of gut microbiota in diabetes pathology. Consequently, rat models have increasingly been employed to elucidate the role and function of the gut microbiota in the context of T2DM. In this investigation, to closely mimic the abnormal glucose fluctuations commonly managed in clinical diabetes care, we employed a diabetic rat model that exhibited such glucose variability. Such models are invaluable for shedding light on potential therapeutic strategies aimed at combating diabetes. Nevertheless, this study has some limitations. First, we acknowledge the limited scope of the sample size and experimental duration. Furthermore, due to current limitations in measurement technology, the conclusions based on empirical data have not been extensively explored through *in vitro* studies or validated at the molecular level, specifically regarding mRNA expressions related to the identified metabolic pathways. This gap underscores the necessity for future investigations to probe deeper into the mechanisms underlying glycemic variability.

## 5 Conclusion

In conclusion, this study elucidates the hypoglycemic effects of the TCM formula SQ, highlighting its significant impact in reducing blood glucose levels, mitigating inflammatory cytokines, enhancing lipid profiles, and alleviating insulin resistance. Moreover, SQ positively influenced the diabetes model animals by regulating the diversity of intestinal microbiota and augmenting various metabolic pathways. These findings suggest that SQ has the potential to serve as a natural therapeutic agent in diabetes management. Additionally, the outcomes of this study provide a theoretical foundation for the effective use of herbal medicine as a valuable resource in managing T2DM.

## Data Availability

The datasets presented in this study can be found in NCBI (BioProject), accession number: PRJNA1057589, available at https://www.ncbi.nlm.nih.gov/bioproject/?term=PRJNA1057589/ Supplementary Material.

## Abbreviations

T2DM, Type 2 diabetes mellitus; SCFAs, Short-chain fatty acids; TCM, traditional Chinese medicine; SQ, Shenqi compound formula; H&E, Hematoxylin-eosin; TG, triglycerides; TC, total cholesterol; HDL-C, high-density lipoprotein cholesterol; LDL-C, low-density lipoprotein cholesterol; TNF-α, Tumor Necrosis Factor Alpha; IL-6, Interleukin 6; GK, Goto-Kakizaki rats; LABG, the largest amplitude of glycemic excursions; MBG, daily mean blood glucose; SDBG, and the standard deviation of daily average blood glucose; FBG, Fasting Blood Glucose; OGTT, oral glucose tolerance test; FINS, fasting insulin in serum; HOMA-IR, homeostasis model assessment-insulin resistance; LC-MS, liquid chromatography-mass spectrometry; PCA, principal component analysis; OPLS-DA, orthogonal projections to latent structures-discriminant analysis; PCoA, principal Coordinate Analysis; LEfSe, Linear Discriminant Analysis Effect Size; LDA, Linear discriminant analysis.
