# Supplementary material for: Investigation of the hypoglycemic mechanism of the ShenQi compound formula through metabonomics and 16S rRNA sequencing
Source: Front Pharmacol. 2024 Apr 19;15:1349244. doi: 10.3389/fphar.2024.1349244 (PMC11066276; doi:10.3389/fphar.2024.1349244)
Supplement: Supplementary file 1 [file Table1.DOCX]

**Table S1** Detection of differential significant metabolites in hepatic samples.

|  |  |  | CON vs. MOD | | |  |  | SAX vs. MOD | | |  |  | SQ vs. MOD | | |  |
| --- | --- | --- | --- | --- | --- | --- | --- | --- | --- | --- | --- | --- | --- | --- | --- | --- |
| **Metabplites** | **MW [g.mol^-1^]** | **Fold**  **change** | | **VIP** | **Trend** | | **Fold change** | | **VIP** | **Trend** | | **Fold**  **change** | | **VIP** | **Trend** | |
| PPHP | 178.23 | 0.29 | | 1.49 | ↓ | | 1.83 | | 2.04 | ↑ | | 1.76 | | 1.82 | ↑ | |
| Trandolaprilat | 402.5 | 0.72 | | 1.20 | ↓ | | 1.38 | | 2.00 | ↑ | | 1.31 | | 1.48 | ↑ | |
| Glu-Val | 246.26 | 0.39 | | 1.21 | ↓ | | 3.32 | | 2.06 | ↑ | | 3.85 | | 1.63 | ↑ | |
| Ercalcitriol | 428.6 | 0.00 | | 1.97 | ↓ | | 0.65 | | 2.18 | ↑ | | 1.66 | | 1.77 | ↑ | |
| Farnesylcysteine | 325.5 | 0.54 | | 1.81 | ↓ | | 1.24 | | 1.99 | ↑ | | 1.39 | | 1.96 | ↑ | |
| Cholesterol | 386.7 | 0.02 | | 1.76 | ↓ | | 5.95 | | 2.35 | ↑ | | 6.39 | | 2.17 | ↑ | |
| 2-Hydroxy-6-tridecylbenzoic acid | 320.5 | 0.33 | | 1.83 | ↓ | | 1.98 | | 2.11 | ↑ | | 1.66 | | 1.54 | ↑ | |
| 5,6-Dihydroxyprostaglandin F1a | 388.5 | 4.10 | | 1.68 | ↑ | | 0.61 | | 1.92 | ↓ | | 0.33 | | 2.21 | ↓ | |
| Citreoviridin D | 416.5 | 1.39 | | 1.13 | ↑ | | 0.67 | | 2.60 | ↓ | | 0.73 | | 1.67 | ↓ | |
| Chinenoside VI | 903 | 0.16 | | 1.63 | ↓ | | 2.42 | | 2.31 | ↑ | | 2.39 | | 1.99 | ↑ | |
| Simulansine | 327.4 | 2.40 | | 1.45 | ↑ | | 0.58 | | 2.09 | ↓ | | 0.44 | | 2.11 | ↓ | |
| LysoPC(20:3(5Z,8Z,11Z)) | 545.7 | 1.70 | | 1.42 | ↑ | | 0.61 | | 2.44 | ↓ | | 0.43 | | 2.38 | ↓ | |
| (3b,9R)-5-Megastigmene-3,9-diol 9 | 506.6 | 2.60 | | 1.67 | ↑ | | 0.58 | | 2.49 | ↓ | | 0.57 | | 2.00 | ↓ | |
| Maraviroc | 513.7 | 3.29 | | 1.57 | ↑ | | 0.44 | | 2.17 | ↓ | | 0.14 | | 2.39 | ↓ | |
| Retapamulin | 517.799 | 1.67 | | 1.61 | ↑ | | 0.71 | | 2.05 | ↓ | | 0.51 | | 2.37 | ↓ | |
| 3-(Acetyloxy)-2-hydroxypropyl octadecanoate | 400.6 | 13.04 | | 1.87 | ↑ | | 0.63 | | 1.88 | ↓ | | 0.44 | | 2.02 | ↓ | |
| (2R,6x)-7-Methyl-3-methylene-1,2,6,7-octanetetrol 2 | 366.4 | 1.39 | | 1.43 | ↑ | | 0.84 | | 1.95 | ↓ | | 0.77 | | 1.73 | ↓ | |
| Erinapyrone A | 142.15 | 0.77 | | 1.28 | ↓ | | - | | - | - | | 1.15 | | 1.69 | ↑ | |
| Galabiosylceramide (d18:1/16:0) | 862.2 | 3.09 | | 1.69 | ↑ | | - | | - | - | | 0.57 | | 1.81 | ↓ | |
| (22E,24R)-Stigmasta-4,22-diene-3,6-dione | 424.7 | 0.16 | | 1.22 | ↓ | | - | | - | - | | 2.18 | | 1.59 | ↑ | |
| p-Cresol | 108.14 | 0.76 | | 1.39 | ↓ | | - | | - | - | | 1.23 | | 1.70 | ↑ | |
| Benzofuran | 118.13 | 0.82 | | 1.26 | ↓ | | - | | - | - | | 1.22 | | 1.86 | ↑ | |
| m-Coumaric acid | 164.16 | 0.81 | | 1.33 | ↓ | | - | | - | - | | 1.21 | | 1.86 | ↑ | |
| Licoriphenone | 372.4 | 0.59 | | 1.31 | ↓ | | - | | - | - | | 1.34 | | 1.67 | ↑ | |
| Kiwiionoside | 406.5 | 0.59 | | 1.22 | ↓ | | - | | - | - | | 1.42 | | 1.56 | ↑ | |
| Armillane | 420.5 | 0.51 | | 1.33 | ↓ | | - | | - | - | | 2.65 | | 1.95 | ↑ | |
| Leucyl-Arginine | 287.36 | 0.16 | | 1.42 | ↓ | | - | | - | - | | 9.69 | | 2.10 | ↑ | |
| (3b,20R,22R)-3,20,27-Trihydroxy-1-oxowitha-5,24-dienolide 3-glucoside | 634.8 | 0.27 | | 1.33 | ↓ | | - | | - | - | | 2.96 | | 1.80 | ↑ | |
| Triamcinolone | 394.4 | 0.36 | | 1.31 | ↓ | | - | | - | - | | 2.41 | | 1.60 | ↑ | |
| L-Carnitine | 161.2 | 0.81 | | 1.48 | ↓ | | - | | - | - | | 1.21 | | 1.73 | ↑ | |
| Torvoside E | 770.9 | 0.38 | | 1.22 | ↓ | | - | | - | - | | 2.65 | | 2.35 | ↑ | |
| Panaquinquecol 6 | 318.4 | 0.39 | | 1.15 | ↓ | | - | | - | - | | 2.36 | | 2.01 | ↑ | |
| Camellenodiol | 442.7 | 0.01 | | 2.02 | ↓ | | - | | - | - | | 5.54 | | 2.15 | ↑ | |
| Camelledionol | 440.7 | 0.02 | | 2.01 | ↓ | | - | | - | - | | 3.18 | | 1.89 | ↑ | |
| Thr Asp Arg Phe | 7054 | 0.43 | | 1.11 | ↓ | | - | | - | - | | 1.75 | | 1.83 | ↑ | |
| 4alpha-Formyl-4beta-methyl-5alpha-cholesta-8,24-dien-3beta-ol | 426.7 | 0.20 | | 1.94 | ↓ | | - | | - | - | | 1.76 | | 1.81 | ↑ | |
| 4,4-Dimethylcholesta-8,14,24-trienol | 410.7 | 0.15 | | 1.73 | ↓ | | - | | - | - | | 1.89 | | 1.90 | ↑ | |
| Hydrocinnamic acid | 150.17 | 0.66 | | 1.62 | ↓ | | - | | - | - | | 1.28 | | 2.01 | ↑ | |
| Asparaginyl-Hydroxyproline | 245.23 | 4.81 | | 1.63 | ↑ | | - | | - | - | | 0.28 | | 2.21 | ↓ | |
| 13-Demethyl tacrolimus | 790 | 2.29 | | 1.87 | ↑ | | - | | - | - | | 0.54 | | 2.39 | ↓ | |
| Vinaginsenoside R2 | 829 | 2.82 | | 1.84 | ↑ | | - | | - | - | | 0.51 | | 2.24 | ↓ | |
| Hovenidulcioside B1 | 855 | 0.42 | | 1.65 | ↓ | | - | | - | - | | 1.62 | | 2.01 | ↑ | |
| Jubanine C | 665.8 | 3.06 | | 1.19 | ↑ | | - | | - | - | | 0.33 | | 1.60 | ↓ | |
| N,O-Didesmethylvenlafaxine | 249.35 | 4.19 | | 1.68 | ↑ | | - | | - | - | | 0.48 | | 2.04 | ↓ | |
| Mabioside D | 799 | 3.79 | | 1.55 | ↑ | | - | | - | - | | 0.31 | | 2.09 | ↓ | |
| Azaspiracid 2 | 856.1 | 2.55 | | 1.47 | ↑ | | - | | - | - | | 0.38 | | 1.89 | ↓ | |
| Aspartyl-Arginine | 289.29 | 5.73 | | 1.56 | ↑ | | - | | - | - | | 0.35 | | 1.98 | ↓ | |
| Perindoprilat | 340.4 | 0.59 | | 1.53 | ↓ | | - | | - | - | | 1.55 | | 1.91 | ↑ | |
| Yucalexin A16 | 302.5 | 0.40 | | 1.42 | ↓ | | - | | - | - | | 1.97 | | 2.16 | ↑ | |
| MG(0:0/20:3(11Z,14Z,17Z)/0:0) | 380.6 | 1.55 | | 1.49 | ↑ | | - | | - | - | | 0.68 | | 1.64 | ↓ | |
| CPA(18:1(11Z)/0:0) | 418.5 | 0.75 | | 1.69 | ↓ | | - | | - | - | | 1.18 | | 1.76 | ↑ | |
| 2,3-bis (4-Hydroxyphenyl) Propionitrile | 239.27 | 1.26 | | 1.37 | ↑ | | - | | - | - | | 0.86 | | 1.90 | ↓ | |
| PC(18:1(9E)/0:0) | 521.7 | 1.28 | | 1.34 | ↑ | | - | | - | - | | 0.82 | | 1.67 | ↓ | |
| PE(20:3(5Z,8Z,11Z)/22:0) | 826.2 | 0.23 | | 1.22 | ↓ | | - | | - | - | | 3.86 | | 2.47 | ↑ | |
| 5,7alpha-Dihydro-1,4,4,7a-tetramethyl-4H-indene | 174.28 | 8.27 | | 1.21 | ↑ | | - | | - | - | | 0.07 | | 1.59 | ↓ | |
| beta-Citraurol | 434.7 | 0.36 | | 1.85 | ↓ | | - | | - | - | | 1.70 | | 1.82 | ↑ | |
| LysoPC(17:0) | 509.7 | 0.51 | | 1.92 | ↓ | | - | | - | - | | 1.27 | | 1.99 | ↑ | |
| Saccharopine | 276.29 | 2.83 | | 1.87 | ↑ | | - | | - | - | | 0.63 | | 2.18 | ↓ | |
| Linatine | 259.26 | 3.37 | | 1.64 | ↑ | | - | | - | - | | 0.53 | | 1.67 | ↓ | |
| Thr Glu Gly | 305.28 | 0.29 | | 1.29 | ↓ | | - | | - | - | | 1.43 | | 2.03 | ↑ | |
| PE(22:4(7Z,10Z,13Z,16Z)/P-18:1(11Z)) | 778.1 | 1.75 | | 1.35 | ↑ | | - | | - | - | | 0.71 | | 1.72 | ↓ | |
| MG(16:1(9Z)/0:0/0:0) | 328.5 | 0.26 | | 1.20 | ↓ | | - | | - | - | | 0.68 | | 1.42 | ↓ | |
| PC(19:0/0:0) | 537.7 | 0.78 | | 1.29 | ↓ | | - | | - | - | | 1.35 | | 1.97 | ↑ | |
| Dehydrocurdione | 234.33 | 0.55 | | 1.26 | ↓ | | - | | - | - | | 1.65 | | 1.52 | ↑ | |
| LysoPE(0:0/22:0) | 537.7 | 0.45 | | 1.41 | ↓ | | - | | - | - | | 1.43 | | 2.14 | ↑ | |
| Falcarindiol | 260.399 | 0.56 | | 1.71 | ↓ | | - | | - | - | | 1.27 | | 1.49 | ↑ | |
| Kinetin | 215.21 | 2.20 | | 1.43 | ↑ | | - | | - | - | | 0.55 | | 1.84 | ↓ | |
| Pyrocoll | 186.17 | 0.40 | | 1.20 | ↓ | | - | | - | - | | 1.51 | | 1.90 | ↑ | |
| 2-Hydroxymyristoylcarnitine | 387.6 | - | | - | - | | 1.51 | | 2.01 | ↑ | | 1.64 | | 1.80 | ↑ | |
| Dinorcapsaicin | 277.36 | - | | - | - | | 1.48 | | 2.03 | ↑ | | 1.60 | | 1.91 | ↑ | |
| 1-Hexanol arabinosylglucoside | 396.4 | - | | - | - | | 0.68 | | 2.16 | ↓ | | 0.58 | | 2.14 | ↓ | |
| MG(0:0/14:0/0:0) | 302.4 | - | | - | - | | 0.81 | | 2.29 | ↓ | | 0.83 | | 1.69 | ↓ | |
| Corosin | 518.7 | - | | - | - | | 0.66 | | 2.41 | ↓ | | 0.74 | | 1.89 | ↓ | |
| DG(16:0/20:5(5Z,8Z,11Z,14Z,17Z)/0:0) | 614.9 | - | | - | - | | 0.48 | | 2.21 | ↓ | | 0.50 | | 1.77 | ↓ | |
| DG(20:5(5Z,8Z,11Z,14Z,17Z)/22:4(7Z,10Z,13Z,16Z)/0:0) | 691 | - | | - | - | | 0.56 | | 2.41 | ↓ | | 0.52 | | 1.82 | ↓ | |
| Isoachifolidiene | 344.4 | - | | - | - | | 1.31 | | 2.19 | ↑ | | 1.29 | | 1.73 | ↑ | |
| 11-Hydroxyprogesterone 11-glucuronide | 506.6 | - | | - | - | | 8.18 | | 2.25 | ↑ | | 8.42 | | 1.88 | ↑ | |
| 1-Isothiocyanato-2-phenylethane | 163.24 | - | | - | - | | 1.25 | | 1.93 | ↑ | | 1.23 | | 1.75 | ↑ | |
| Riboflavin cyclic-4',5'-phosphate | 438.3 | - | | - | - | | 1.28 | | 1.91 | ↑ | | 1.27 | | 1.81 | ↑ | |
| Flavin adenine dinucleotide (FAD) | 785.5 | - | | - | - | | 1.21 | | 1.95 | ↑ | | 1.24 | | 1.99 | ↑ | |
| N2-Succinoylarginine | 274.27 | - | | - | - | | 1.32 | | 2.52 | ↑ | | 1.50 | | 2.25 | ↑ | |
| Retinyl beta-glucuronide | 462.6 | - | | - | - | | 3.67 | | 1.85 | ↑ | | 3.77 | | 1.88 | ↑ | |
| Auraptene | 298.4 | - | | - | - | | 0.40 | | 2.10 | ↓ | | 0.31 | | 1.92 | ↓ | |
| alpha-[3-[(Hydroxymethyl)nitrosoamino]propyl]-3-pyridinemethanol | 225.24 | - | | - | - | | 0.55 | | 1.94 | ↓ | | 0.51 | | 1.82 | ↓ | |
| 1-(9Z-tetradecenoyl)-glycero-3-phosphate | 380.4 | - | | - | - | | 2.36 | | 2.08 | ↑ | | 2.15 | | 1.69 | ↑ | |
| 4-Epiisoinuviscolide | 248.32 | - | | - | - | | 1.11 | | 1.86 | ↑ | | 1.24 | | 2.27 | ↑ | |
| LysoPE(0:0/20:4(8Z,11Z,14Z,17Z)) | 501.6 | - | | - | - | | 1.14 | | 1.91 | ↑ | | 1.33 | | 2.32 | ↑ | |
| 24-Acetyl- 25-cinnamoylvulgaroside | 608.8 | - | | - | - | | 0.44 | | 2.29 | ↓ | | 0.16 | | 2.43 | ↓ | |
| Calcidiol | 400.6 | - | | - | - | | 4.08 | | 2.25 | ↑ | | 5.48 | | 1.77 | ↑ | |
| 22-Angeloyltheasapogenol A | 588.8 | - | | - | - | | 1.52 | | 2.00 | ↑ | | 2.00 | | 1.88 | ↑ | |
| Glutaminyl-Tryptophan | 332.35 | - | | - | - | | 0.58 | | 2.13 | ↓ | | 0.59 | | 1.86 | ↓ | |
| Calcidiol | 400.6 | - | | - | - | | 4.08 | | 2.25 | ↑ | | 5.48 | | 1.77 | ↑ | |

Note:The bar indicates that there is no significant change in metabolite.
